# Why a Right to Health Makes No Sense, and What Does

**DOI:** 10.1089/heq.2019.0116

**Published:** 2020-06-12

**Authors:** Robert A. Hahn, Carles Muntaner

**Affiliations:** ^1^ Department of Anthropology, Emory University, Atlanta, Georgia, USA.; ^2^ Dalla Lana School of Public Health, Social Equity and Health, University of Toronto, Toronto, Canada.

**Keywords:** equity, social determinants of health, right to health

## Abstract

There is a widely held belief in a universal right to the highest attainable standard of health. This essay shows how this right is conceptually unclear, unattainable, and a distraction from a more concrete and attainable right: a right to equitable access to available resources for health (RARH), including equitable access to the social determinants of health. It clarifies conceptual and theoretical issues in the RARH: its underlying theory rooted in historical, economic, and axiological rationales; its concept of component resources and their availability, equity, sustainability; and the redistribution of wealth and power, metrics, and ethics. The advancement of global health equity requires explicit theorizing of what underlies a right to health. The right to the highest attainable standard of health fails in this regard. The RARH provides a desirable, actionable, and measurable foundation for global health equity.

## Introduction

There is a widely held belief in a universal right to health (i.e., RTH) that some claim is a right to the highest attainable standard of health. This goal could be referred to as optimal health.

The World Health Organization (WHO) Constitution of 1946 proclaimed:

“The States Parties to this Constitution declare, in conformity with the Charter of the United Nations, that the following principles are basic to the happiness, harmonious relations and security of all peoples:

Health is a state of complete physical, mental and social well-being and not merely the absence of disease or infirmity.

The enjoyment of the highest attainable standard of health is one of the fundamental rights of every human being without distinction of race, religion, political belief, economic or social condition.”

WHO Constitution 1946

If “enjoyment of…. is one of the fundamental rights…,” then at least some of us must have achieved this state, and the Constitution proposes that all humans should be able to reach this state—it is their right. The RTH is repeatedly cited by WHO leaders and others^1–3^ and is widely used to justify programs and policies.^3^ Some documents note that the RTH includes not only health itself but also access to the social determinants of health (SDOH).^4,5^ Since all of humanity is promised optimal health, the RTH implies health equity—all of humanity achieving this optimal state. Some claim that aspects of the RTH are legally enforceable.^1,2^ While critiques of the RTH have been advanced,^4,6–9^ they have not dampened widespread conceptualization, endorsement, and commitment to this right.

In this study, it is argued that this so-called right is conceptually unclear, impossible to fulfill, and that it thus creates false expectations. While rights may be framed aspirationally, their conceptual components should be understandable and steps toward them practical and measurable. Attention to RTH diverts attention from a right that is clearer, plausible, and a desirable, challenging, but achievable, goal—namely, a right to equitable access to available resources for health (i.e., RARH). Problems in the idea of a right to health are explored and the elements of a preferable right are presented. The commonly expressed right, while emotionally appealing, is mistakenly formulated and seriously misleading. We share a strong moral commitment to the equitable distribution of health resources, including social determinants, to all people on earth, now and in the future, but we believe that the idea of an RTH misconceives the goals and means of achieving this end.

Given the large extent of poverty and poor health in the world, it is critical to clarify what all of humanity has a right to. While the world population living on $1.90 a day has fallen dramatically in the past two decades, still, in 2015, 736 million people worldwide (10% of the world population) lived at this level, and half of the world's population lived on <$5.50 a day.^10^ In 2015, in Sierra Leone, male life expectancy at birth was 51 years and female life expectancy 52 years—the lowest life expectancies in the world. In Hong Kong, males had the highest male life expectancy in the world—81 years, while females had a life expectancy of 87 years. The WHO announced in 2008 the possibility of closing the gap in a generation,^11^ and the World Bank has proposed the elimination of extreme poverty and boosting shared prosperity of the bottom 40 percent of populations in every country.^10(p.ix)^ These are projects of unprecedented human scale.

## Why RTH Makes No Sense

### The goal is ill-defined and unachievable

The 1978 Alma-Ata International Conference on Primary Health Care defined health to include “a state of complete…mental and social well-being….”^12^ This famous definition expanded the conceptualization of health beyond physiological conditions to include a person's mental well-being and social environment. However, the definition was aspirational. Who has achieved this complete state and to what might this state objectively refer? In addition, the health that is the goal of the RTH is likely to be heterogeneous—different for every human being.^13^ What does it mean for someone with a disability, a marathoner, a sprinter, a computer whiz, a mystic, a mechanic, or a president to have optimal health? In large part, health is the ability to respond to one's environment effectively, and environments vary and change. Thus, what optimal health might be is unclear. If the goal is ill-defined, it cannot be measured, approaches to it cannot be evaluated, and its achievement cannot be monitored.

### Resources may not be available

Even if the concept of optimal health was clearer, what resources would it take to move an individual from a current state to that optimal state? Resources include not only materials and products but also knowledge and skills to recognize and treat the plethora of human ills. There are many conditions for which prevention or cure is not yet known, and there are many known conditions that are currently very expensive to prevent or cure. Can optimal health be achieved for anyone? In addition, what about attaining optimal health for the billions of humans with diverse and substantially greater needs? Do knowledge and material resources to do this exist?

The notion of available resources is problematic. When a nation claims to have no funds to address poverty while allocating >50% of its budget to the military and granting large tax breaks to the wealthy, talk of commitment to health for all appears empty. Resources said to be available are, to a large extent, a matter of priorities and will. In many wealthy nations, systems of taxation have moved toward increasing benefits for the wealthiest, providing less and less for those with greatest needs.^14^ In contrast, several Nordic and other European nations have high net rates of taxation on those with the highest income, fostering the redistribution of wealth in their nations.^15^ It is estimated that it would take <$1.4 trillion annually to meet the United Nations Sustainable Development Goals of providing health services, education, clean water, and social services in the world's poorest countries—less than 2% of the world's gross domestic product (GDP) and a fraction of the annual world's expenditures on arms.^16^ However, even if success in moving those in poverty to a state of optimal health were to be achieved, there are billions of others in lesser forms of poverty who nevertheless are also in need of health and societal interventions,^17^ and then, there are the rest of us who are not only relatively wealthy—the global upper and middle classes,^18^ but most likely also not in optimal health.

What is the merit of professing a right whose goals are unclear and whose achievement is implausible if not impossible? While much public health good may have been created under the auspices of the right to health, these successes do not justify the underlying logic of this banner. The underlying conceptualization of the RTH diverts attention from potentially more effective interventions.

## Basic Issues in the RARH

A right proposed here is within human capacity to provide. A right to equitable access is an advance over the RTH because it is independent of the resources available. Whatever resources for health are available, whether few or many, can be equitably divided.

Why pursue this alternative RARH? Our primary and fundamental motivation for a commitment of health equity is a moral matter of justice.^19^ The moral obligation is to the well-being of each person on earth so that even if it were costly to assure equitable resources for all, it would be a global responsibility to work toward fulfillment of that right.

There is a related matter of justice for pursuing equitable access. It is likely that the wealth of the high-income Western world is the result of its longstanding exploitation of the low-income non-Western world, beginning with European explorations of the globe in the 15th century, followed by colonial empires and slavery, and continuing in multiple, if more muted, forms.^20,21^ Global health equity—the right for all to equitable access to available global resources for health—would thus be partial compensation for centuries of exploitation and deprivation. This position may underlie the excessively cautious statement of the 2008 WHO Commission on SDOH: “The Commission takes issue with the unequal distribution of social conditions when health suffers as a consequence.”^22^ Within nations such as the United States, similar exploitation, for example, exploitation by whites of black enslaved people and others, should also be grounds for equity if not reparations.^23–26^

There is an economic reason for pursuing equity. There is evidence that inequity is costly in several ways, so its elimination may have economic benefits, perhaps outweighing the costs of elimination.^27,28^ It is difficult, if not impossible, to calculate the fundamental cost of poverty in the suffering of those who experience it.^29^ Among the societal costs of poverty and inequity are the underdeveloped capacities and fulfillment of the populations without resources (of food, shelter, education, civic participation, and so on). They cannot fully participate fruitfully in labor and they do not contribute to taxes that theoretically support public institutions. Moreover, they may consume societal resources, for example, in terms of health care, welfare, and the justice system, even if poverty is not a personal failing, but a consequence of social institutions.

There have been various estimates of the costs of poverty and low levels of education in the United States, in economic terms, and in terms of health.^30–32^ LaViest and colleagues estimate the direct and indirect costs associated with the health and health care of more deprived populations in the United States from 2003 to 2006 to be $1.24 trillion. Mackenbach estimates the value of health inequality-related welfare losses in European nations to be €980 billion per year or 9.4% of GDP.^33^ There are also estimates of the economic benefits of diverse programs for low-income nations that indicate the financial benefit of investing in them.^34^

### What are the resources for health?

The resources for health include not only health care and preventive health care but also the SDOH, such as aging, gender, childhood, social cohesion, neighborhood, education, employment, housing, civic participation, power, transportation, and justice.^35–38^ SDOH may be more powerful in determining health outcomes than health care.^22^ It has been shown that if all youth in the United States were to graduate from high school, more premature mortality would be averted than by the elimination of smoking in the population.^39^ It is unlikely that good health can be achieved for populations if the SDOH are not equitably distributed. Thus, if the RARH is to be guaranteed, equity in access to underlying SDOH must be included among resources for health.

### What is meant by available resources?

Available with what effort—within arm's reach, in the immediate surroundings, anywhere, and with what exertion? Are these resources yet to be discovered and located? Who is doing this work and how are they recompensed? There are other boundary conditions on available resources—for example, given other uses of the same resources—for art, industry, defense, and so on, rather than for health equity. The uses of whatever resources are made available must be prioritized and allocated. How are such decisions made and by whom? Most often, allocations are made by policy makers and lobbyists, and those with money/power make those decisions.^22^

### What are the societal bounds within which equitable access is promised in RARH?

Should equitable access be pursued among the members of communities, nations, or across the globe?^18^ The global equity position is advocated here because of the history noted above, which has produced the inequitable global distribution of resources seen today. This position is consistent with the humanistic ethics according to which health inequities that are deemed unjust and avoidable apply to all humans, not just the population of the United States or other first-world countries.^40,41^

### What is meant by equitable access to those resources?

Should the RARH assure equal allocation of resources for all? Equal, for example, each person gets $1000, is not equitable. What I need to improve my health and what others need are likely to differ substantially. My needs are far fewer than the millions with little food or shelter. In addition, during my lifetime, I have not been deprived of available health care, and I have had access to far more than my share of determinants. Equitable suggests allocation to each according to need, given the limits on resources. Equity may be challenging because interventions needed to treat some conditions may exhaust resources. There must be some process by which allocation and distribution can be limited, that is, rationing—so that all have access. Resource limitations underlie the critical restriction of available resources in the right advocated here. Given the limits on resources, rationing is not an option one can decide to accept or forgo, but unavoidable. Rationing happens, whether one works to control the process, democratically, or just allows it to happen, with others making the decisions. Equitable allocation can and should be a choice. A recent document by Oxfam summarizing a broad array of recent evidence proposes that (at least in the developed world) appropriate collection of taxes from the wealthy and corporations would allow equitable good health for all.^14^

### RARH must be pursued not only for those currently alive but for succeeding generations as well

This commitment requires the sustainability of equity programs and of the resources for such programs, attention to the global environment, renewable resources, and the assurance that human habitation of the earth can fruitfully persist. The WHO report recognized this need.^22^

A major challenge of the RARH is the necessary national and global redistribution of resources and power—that is, the control of resources, particularly considering that social determinants must be included in the equitable distribution. It is implausible that equity could be achieved while the current wealth and power of the wealthy and powerful are maintained. The wealthy and powerful will have to surrender large portions of their wealth and power. Given that these same people are often dominant in politics and in the media, their democratic outvoting will be a challenge. The WHO report hints of this transformation, but does not explicitly name it, vaguely referring to changing the distribution of power within society and global regions, especially in favor of disenfranchised groups and nations.^22^ Nevertheless, movement has been made in a more positive direction, and a number of the wealthy and powerful recognize the benefits or necessity of this redistribution (e.g., The Giving Pledge, including 175 wealthy members, such as Bill and Melinda Gates, Warren Buffet, Ted Turner, and Charles Feeney, who have committed and spent large sums on equity-related projects). In contrast, other wealthy families and organizations actively work to further concentrate their wealth at the expense of others, the environment, and thus future generations. Whether equity can be achieved despite them remains to be seen.^42^ Oxfam advocates equitable taxation as a fundamental element of the solution of global inequity.^43^

Many programs are effective and cost-beneficial in assisting the movement of impoverished populations in poor health into economically improved situations and also produce improved health.^34^ Conditional and unconditional cash transfer programs have demonstrated success in moving low-income populations in many nations to higher levels of education, health, and income.^44^ In conditional programs, low-income households are given money under specified conditions, for example, they send their children to school. In unconditional programs, money is given without such requirements.^45^ Experimental studies among the extremely poor in six countries have demonstrated multiple benefits of multifaceted graduated programs, which include cash transfers and substantial training and guidance.^46^ In high-income nations, for example, Canada and Finland, there is evidence that guaranteed income programs have benefits in terms of employment and health.^47,48^ Guaranteed income programs for low-income families have been tested in the United States and Canada and shown, contrary to expectation, to only minimally reduce recipients' commitment to work.^47^

### Progress toward RARH can be measured

Knowing the available resources for health, their distribution and changes in their distribution among populations can be monitored.^49^ Metrics are available and used to measure and track health equity/health outcomes: GINI ratios^†^ of the distributions of economic and other resources within and among nations; levels of poverty by nation and globally^18^; national and global health levels, for example, mortality or disability-adjusted life years^50^; and the redistribution of resources within and among nations.^51^

In the pursuit of health equity, attention to the voices and participation of those whose lives are the object of improvement are essential.^52^ While scientists, policy makers, and program designers may have findings, statistics, and theories about their lives, it is they who know best the suffering they may endure and what counts as success when interventions are implemented. They are likely to know their needs and how they might be met. Their perception and knowledge are essential to effective program design. Their likely engagement in implemented programs is an added benefit. The ethical approach for researchers and policy makers is thus to involve the persons who are affected by our work.

## Conclusion

In moving toward global public health and justice, it is critical to be clear and practical about ways to achieve our goals. Health and the highest attainable health as human rights fail in this regard. Let us cease to speak and write misleadingly of a RTH or the highest attainable health. The RTH is an illusion and a distraction, as is the highest attainable health. A pragmatic RARH is reasonable. Some polities have achieved this to a greater extent than others, suggesting that such arrangements are possible.^53^ Several Scandinavian nations have focused on equity in resources for social determinants, such as education, as a means of producing health equity.^53^

What does health equity look like for all individuals worldwide? Privilege by class, race, gender, and so on is gone. In the United States, health equity would include the absence of segregated neighborhoods and segregated schools, disproportionate minority contact with the justice system, racial profiling, and the “1%”—societal segments in the highest income percentile whose income is greatly in excess of its population proportion. It would mean the end of homelessness and the availability of affordable housing. It would mean widely accessible public transportation linking heterogeneous communities. It would mean meaningful employment and living wages. Globally, it implies the absence of North and South, high- and low-income nations, and vast gaps in well-being within nations. The RARH provides a clear rationale for this achievable vision.

## Abbreviations Used

RARH: right to equitable access to available resources for health
RTH: right to health
SDOH: social determinants of health
WHO: World Health Organization

## Notes

^†^ The GINI ratio, named after the statistician who devised it, is a statistical measure of the unequal distribution of income in a population; it ranges from 0, indicating total equality, to 1, indicating extreme inequality.
